# Supplementary material for: Expediting evidence synthesis for healthcare decision-making: exploring attitudes and perceptions towards rapid reviews using Q methodology
Source: PeerJ. 2016 Oct 6;4:e2522. doi: 10.7717/peerj.2522 (PMC5068451; doi:10.7717/peerj.2522)
Supplement: Supplemental Information 1 [file peerj-04-2522-s001.docx]

**Q-sort correlation matrices**

The correlation matrix represents all of the meaning and variability found in this study.

|  | **1** | **2** | **3** | **4** | **5** | **6** | **7** | **8** | **9** | **10** | **11** |
| --- | --- | --- | --- | --- | --- | --- | --- | --- | --- | --- | --- |
| **1** | 100 | 13 | -19 | 4 | 12 | -2 | 16 | 9 | -7 | 34 | 18 |
| **2** | 13 | 100 | 40 | 42 | 45 | 37 | 60 | 45 | 48 | 64 | 26 |
| **3** | -19 | 40 | 100 | 43 | 30 | 27 | 39 | 20 | 38 | 36 | 1 |
| **4** | 4 | 42 | 43 | 100 | 25 | 48 | 45 | 32 | 45 | 42 | 7 |
| **5** | 12 | 45 | 30 | 25 | 100 | 23 | 29 | 39 | 37 | 47 | 12 |
| **6** | -2 | 37 | 27 | 48 | 23 | 100 | 26 | 51 | 42 | 38 | 20 |
| **7** | 16 | 60 | 39 | 45 | 29 | 26 | 100 | 44 | 55 | 58 | 20 |
| **8** | 9 | 45 | 20 | 32 | 39 | 51 | 44 | 100 | 48 | 46 | 6 |
| **9** | -7 | 48 | 38 | 45 | 37 | 42 | 55 | 48 | 100 | 43 | 17 |
| **10** | 34 | 64 | 36 | 42 | 47 | 38 | 58 | 46 | 43 | 100 | 20 |
| **11** | 18 | 26 | 1 | 7 | 12 | 20 | 20 | 6 | 17 | 20 | 100 |

Note: A value of 41 represents 0.41 multiplied by 100

***Standard error (SE) of the correlation matrix***

|  | **1** | **2** | **3** | **4** | **5** | **6** | **7** | **8** | **9** | **10** | **11** |
| --- | --- | --- | --- | --- | --- | --- | --- | --- | --- | --- | --- |
| **1** | 100 | 13 | -19 | 4 | 12 | -2 | 16 | 9 | -7 | 34 | 18 |
| **2** | 13 | 100 | 40 | 42 | 45 | 37 | 60 | 45 | 48 | 64 | 26 |
| **3** | -19 | 40 | 100 | 43 | 30 | 27 | 39 | 20 | 38 | 36 | 1 |
| **4** | 4 | 42 | 43 | 100 | 25 | 48 | 45 | 32 | 45 | 42 | 7 |
| **5** | 12 | 45 | 30 | 25 | 100 | 23 | 29 | 39 | 37 | 47 | 12 |
| **6** | -2 | 37 | 27 | 48 | 23 | 100 | 26 | 51 | 42 | 38 | 20 |
| **7** | 16 | 60 | 39 | 45 | 29 | 26 | 100 | 44 | 55 | 58 | 20 |
| **8** | 9 | 45 | 20 | 32 | 39 | 51 | 44 | 100 | 48 | 46 | 6 |
| **9** | -7 | 48 | 38 | 45 | 37 | 42 | 55 | 48 | 100 | 43 | 17 |
| **10** | 34 | 64 | 36 | 42 | 47 | 38 | 58 | 46 | 43 | 100 | 20 |
| **11** | 18 | 26 | 1 | 7 | 12 | 20 | 20 | 6 | 17 | 20 | 100 |

Note: SE calculated as:(( $1/\sqrt{n}$)*100). A value of 41 represents 0.41 multiplied by 100

Orange correlations are statistically significant as they fall between 2 and 2.5 times (SE = 60.3 to 75.4)the standard error.

Yellow correlations trend towards being statistically significant as they come close to being 2 times the standard error (≥50).

**Supplement 7‑15. Factor loadings**

The loadings express the extent to which each Q sort is associated with each factor. Factor loadings in excess of 0.50 (plus or minus) can be considered significant, however.

***Unrotated Q sort factor loading matrix (n=7)***

| **Q sort** | **Factor** | | | | | | | **h^2^** |
| --- | --- | --- | --- | --- | --- | --- | --- | --- |
|  | **A** | **B** | **C** | **D** | **E** | **F** | **G** |  |
| **8174** | 0.13141 | 0.60329 | 0.11441 | 0.29681 | 0.27382 | 0.09923 | -0.12686 | 0.5834 |
| **8176** | 0.78804 | 0.14342 | 0.02191 | -0.06425 | -0.04936 | 0.00146 | 0.04982 | 0.6511 |
| **8185** | 0.45021 | -0.16855 | 0.0131 | -0.37848 | 0.04678 | 0.0084 | 0.38096 | 0.5219 |
| **8186** | 0.60359 | -0.28424 | 0.55147 | -0.05315 | 0.18554 | 0.08993 | 0.06393 | 0.7986 |
| **8194** | 0.53428 | 0.00884 | -0.11966 | 0.05231 | 0.1428 | -0.02699 | 0.07705 | 0.3296 |
| **8198** | 0.55526 | -0.38279 | 0.08575 | 0.46752 | -0.05177 | 0.38573 | 0.29049 | 0.9167 |
| **8199** | 0.72566 | 0.00659 | 0.06709 | -0.18614 | 0.08849 | 0.19419 | -0.28389 | 0.6918 |
| **8200** | 0.6173 | -0.08837 | -0.17681 | 0.15103 | 0.10109 | 0.07367 | 0.01845 | 0.4589 |
| **8215** | 0.67288 | -0.22546 | -0.02935 | -0.04948 | 0.04534 | 0.15306 | -0.08066 | 0.5389 |
| **8221** | 0.80283 | 0.26536 | 0.06865 | 0.08657 | 0.14305 | -0.10885 | 0.03971 | 0.761 |
| **8223** | 0.25014 | 0.19806 | 0.09914 | 0.04339 | -0.29969 | 0.17698 | -0.12329 | 0.2498 |
| **Eigenvalues** | 3.8667 | 0.8086 | 0.3908 | 0.5287 | 0.2675 | 0.2771 | 0.3623 | -- |
| **Total Variance** | 35.1518 | 7.3509 | 3.5527 | 4.8064 | 2.4318 | 2.5191 | 3.2936 | **59.1064** |

Green shading indicates significant by the Fuerntratt criterion (1)

h^2^ = common variance, or communality measure explains how much how much a particular Q sort holds in common with all the other Q sorts in the study group. A high communality signals that the Q sort is typical or highly representative of the group as a whole, a low communality that it is atypical. (Watts, Simon; Stenner, Paul (2012-03-19). Doing Q Methodological Research (Kindle Location 2489). SAGE Publications. Kindle Edition.)

**Rotated Q Sort factor loading matrix (varimax rotation) (n=7)**

| **Q sort** | **Factor** | | | | | | | **h^2^** |
| --- | --- | --- | --- | --- | --- | --- | --- | --- |
|  | **A** | **B** | **C** | **D** | **E** | **F** | **G** |  |
| **8174** | 0.11802 | 0.73443 | -0.00557 | 0.00053 | 0.11949 | -0.03247 | -0.12104 | 0.5834 |
| **8176** | 0.63649 | 0.10406 | 0.2052 | -0.05377 | 0.32896 | 0.08396 | 0.27366 | 0.651 |
| **8185** | 0.30617 | -0.20014 | 0.19695 | 0.01825 | -0.02403 | 0.07102 | 0.58597 | 0.5219 |
| **8186** | 0.28805 | 0.00358 | 0.77397 | 0.01503 | 0.07067 | 0.25821 | 0.21153 | 0.7986 |
| **8194** | 0.52333 | 0.08742 | 0.07885 | -0.01093 | 0.00667 | 0.14315 | 0.14577 | 0.3295 |
| **8198** | 0.31271 | -0.0484 | 0.22382 | 0.00066 | 0.12145 | 0.86398 | 0.07201 | 0.9167 |
| **8199** | 0.5944 | 0.04992 | 0.36496 | 0.31377 | 0.31047 | -0.00293 | 0.08977 | 0.692 |
| **8200** | 0.60049 | 0.03266 | 0.07007 | 0.05938 | 0.05857 | 0.28623 | 0.05903 | 0.4589 |
| **8215** | 0.56684 | -0.12485 | 0.2791 | 0.19298 | 0.15661 | 0.22561 | 0.10695 | 0.5388 |
| **8221** | 0.69778 | 0.33089 | 0.245 | -0.15036 | 0.20115 | 0.06652 | 0.19274 | 0.761 |
| **8223** | 0.09481 | 0.08139 | 0.03328 | 0.01357 | 0.47907 | 0.05718 | -0.01276 | 0.2499 |
| **Eigenvalues** | 2.4985 | 0.7355 | 1.0133 | 0.1655 | 0.5372 | 0.9873 | 0.5644 | -- |
| **Total Variance** | 22.7136 | 6.6864 | 9.2118 | 1.5045 | 4.8836 | 8.9755 | 5.1309 | **59.1064** |

Green shading indicates significant by the Fuerntratt criterion (1)

**Unrotated Q sort factor loading matrix (n=3)**

|  | **A** | **B** | **C** | **h^2^** |
| --- | --- | --- | --- | --- |
| **8174** | 0.13141 | 0.60329 | 0.11441 | 0.3944 |
| **8176** | 0.78804 | 0.14342 | 0.02191 | 0.6421 |
| **8185** | 0.45021 | -0.16855 | 0.0131 | 0.2313 |
| **8186** | 0.60359 | -0.28424 | 0.55147 | 0.7492 |
| **8194** | 0.53428 | 0.00884 | -0.11966 | 0.2999 |
| **8198** | 0.55526 | -0.38279 | 0.08575 | 0.4622 |
| **8199** | 0.72566 | 0.00659 | 0.06709 | 0.5311 |
| **8200** | 0.6173 | -0.08837 | -0.17681 | 0.4202 |
| **8215** | 0.67288 | -0.22546 | -0.02935 | 0.5045 |
| **8221** | 0.80283 | 0.26536 | 0.06865 | 0.7196 |
| **8223** | 0.25014 | 0.19806 | 0.09914 | 0.1116 |
| **Eigenvalues** | 3.8667 | 0.8086 | 0.3908 | -- |
| **Total Variance** | 35.1518 | 7.3509 | 3.5527 | **46.05** |

Green shading indicates significant by the Fuerntratt criterion (1)

**Rotated Q Sort factor loading matrix (varimax rotation) (n=3)**

|  | **A** | **B** | **C** | **h^2^** |
| --- | --- | --- | --- | --- |
| **8174** | -0.04896 | 0.61659 | -0.10834 | 0.3943 |
| **8176** | 0.63758 | 0.4089 | 0.26143 | 0.642 |
| **8185** | 0.40766 | 0.01062 | 0.25489 | 0.2313 |
| **8186** | 0.29542 | 0.11777 | 0.80504 | 0.7493 |
| **8194** | 0.51352 | 0.15932 | 0.10374 | 0.2999 |
| **8198** | 0.49924 | -0.12246 | 0.44492 | 0.4622 |
| **8199** | 0.58604 | 0.27841 | 0.33192 | 0.5311 |
| **8200** | 0.62939 | 0.08502 | 0.12951 | 0.4201 |
| **8215** | 0.62862 | 0.0252 | 0.32963 | 0.5045 |
| **8221** | 0.60607 | 0.5363 | 0.25441 | 0.7196 |
| **8223** | 0.13065 | 0.29252 | 0.09479 | 0.1117 |
| **Eigenvalues** | 2.6944 | 1.0603 | 1.3113 | -- |
| **Total Variance** | 24.4945 | 9.6391 | 11.9209 | **46.0545** |

Green shading indicates significant by the Fuerntratt criterion (1)

**Supplement 7‑16. Factor scores**

**Rank Statement Scores for Factors A, B and C**

| **Statement #** | **Factors** | | | | | |
| --- | --- | --- | --- | --- | --- | --- |
|  | **A** | | **B** | | **C** | |
|  | **Z-Score** | **Rank** | **Z-Score** | **Rank** | **Z-Score** | **Rank** |
| 1 | 0.486 | 16 | 0.621 | 13 | 0 | 32 |
| 2 | -0.783 | 38 | 0.843 | 9 | -2.059 | 50 |
| 3 | -0.262 | 32 | 2.463 | 1 | 0.686 | 18 |
| 4 | -0.861 | 40 | 1.664 | 3 | 0 | 32 |
| 5 | -0.989 | 43 | -1.242 | 45 | -0.686 | 42 |
| 6 | 0.523 | 15 | 0.843 | 9 | 0.686 | 18 |
| 7 | -2.135 | 49 | -2.263 | 49 | -2.059 | 50 |
| 8 | 0.059 | 27 | -1.642 | 47 | 0.686 | 18 |
| 9 | 0.297 | 21 | 1.242 | 5 | 0 | 32 |
| 10 | 1.869 | 1 | 1.442 | 4 | 0 | 32 |
| 11 | -0.893 | 41 | 0.621 | 13 | 0.686 | 18 |
| 12 | -1.943 | 48 | 0.421 | 18 | -1.373 | 48 |
| 13 | 0.339 | 19 | 1.021 | 6 | 2.059 | 2 |
| 14 | 0.674 | 12 | 0.2 | 25 | 1.373 | 8 |
| 15 | -0.952 | 42 | 0 | 30 | -0.686 | 42 |
| 16 | 0.15 | 26 | 0.2 | 25 | -0.686 | 42 |
| 17 | -0.514 | 37 | 0 | 30 | 1.373 | 8 |
| 18 | 1.026 | 8 | 0 | 30 | 0 | 32 |
| 19 | -1.023 | 44 | -2.463 | 50 | -0.686 | 42 |
| 20 | 1.55 | 4 | -0.222 | 35 | 0 | 32 |
| 21 | 1.295 | 5 | 0.621 | 13 | 0.686 | 18 |
| 22 | 0.684 | 11 | 0.2 | 25 | 0 | 32 |
| 23 | 0.211 | 25 | 0.2 | 25 | -0.686 | 42 |
| 24 | 0.298 | 20 | 0.421 | 18 | 1.373 | 8 |
| 25 | 1.246 | 6 | 0.2 | 25 | 0 | 32 |
| 26 | 0.011 | 28 | -1.242 | 45 | 0.686 | 18 |
| 27 | 0.466 | 18 | -0.821 | 42 | 1.373 | 8 |
| 28 | 0.535 | 14 | -1.842 | 48 | 2.059 | 2 |
| 29 | 0.484 | 17 | 0 | 30 | 0 | 32 |
| 30 | -0.148 | 30 | 0.4 | 19 | 0 | 32 |
| 31 | 0.296 | 22 | -0.421 | 37 | -0.686 | 42 |
| 32 | 0.866 | 9 | 0.421 | 18 | 0.686 | 18 |
| 33 | 0.269 | 23 | -0.843 | 43 | 1.373 | 8 |
| 34 | -0.393 | 33 | -0.621 | 40 | -1.373 | 48 |
| 35 | 0.6 | 13 | -0.621 | 40 | 0 | 32 |
| 36 | 0.738 | 10 | 0.843 | 9 | 0 | 32 |
| 37 | -0.462 | 34 | 0.421 | 18 | -0.686 | 42 |
| 38 | -2.149 | 50 | -0.222 | 35 | -1.373 | 48 |
| 39 | 1.173 | 7 | -0.621 | 40 | 0.686 | 18 |
| 40 | -1.53 | 47 | 0.421 | 18 | -0.686 | 42 |
| 41 | 1.837 | 2 | -0.222 | 35 | 1.373 | 8 |
| 42 | -0.856 | 39 | -1.642 | 47 | -0.686 | 42 |
| 43 | -1.396 | 45 | -0.821 | 42 | -1.373 | 48 |
| 44 | -0.505 | 36 | -0.2 | 32 | 0 | 32 |
| 45 | 1.826 | 3 | 0.821 | 10 | 0 | 32 |
| 46 | -0.48 | 35 | 0 | 30 | 0.686 | 18 |
| 47 | -1.412 | 46 | 1.842 | 2 | -1.373 | 48 |
| 48 | -0.118 | 29 | -0.421 | 37 | -0.686 | 42 |
| 49 | 0.235 | 24 | -0.2 | 32 | 0.686 | 18 |
| 50 | -0.237 | 31 | 0.2 | 25 | -1.373 | 48 |

**Normalized Factor Scores for Factors A, B and C**

**Factor A**

| **#** | **Statements** | **Z-Score** |
| --- | --- | --- |
| 10 | All evidence synthesis products, including rapid reviews, SRs, or HTAs, can be conducted very well or very poorly. | 1.869 |
| 41 | A well-conducted rapid review may produce better evidence than a poorly conducted systematic review. | 1.837 |
| 45 | A good quality review of evidence is determined by the methods used, not by the speed at which it is completed. | 1.826 |
| 20 | Rapid reviews and all other evidence synthesis products hold the same value as long as they retain the core value of being transparent in conduct, include the highest quality evidence available and present results with a qualification on the strength of evidence. | 1.55 |
| 21 | Appropriateness of a rapid review varies with the type of decision being made, and any financial, legal or other important contextual facets tied to the decision. | 1.295 |
| 25 | It is important to have minimum standards for the reporting of rapid reviews (e.g., a PRISMA-RR). | 1.246 |
| 39 | Achieving a precise estimate of effect (from a SR) may not inform the decision-at-hand any better than a general estimate of effect (produced by a rapid review). | 1.173 |
| 18 | Using rapid reviews to inform decisions is better than using no evidence at all. | 1.026 |
| 32 | Rapid reviews can be timely and valid, even when methodological concessions are made. | 0.866 |
| 36 | The results from a systematic review may not differ from those of a rapid review, but more research is needed to support this theory and quantify why results may be the same or different. | 0.738 |
| 22 | My confidence in a rapid review is impacted by which methods are tailored to speed up the review process. | 0.684 |
| 14 | Rapid reviews meet the needs of knowledge users. | 0.674 |
| 35 | Rapid reviews are not a unique methodology, they are simply a variation of a systematic review that can fall anywhere on the continuum of evidence synthesis methods. | 0.6 |
| 28 | Knowledge users don't always need all of the evidence, they just need the best evidence to support their decision, and what is ‘best evidence' is specific to the knowledge user. | 0.535 |
| 6 | Rapid reviews mean different things to different people. | 0.523 |
| 1 | The evidence from rapid reviews is good enough to inform low-risk, emergent policy or decision-making needs when the alternative is the use of no evidence. | 0.486 |
| 29 | Knowledge users do not fully understand the implications of streamlining evidence synthesis methods to produce a more timely evidence product. | 0.484 |
| 27 | The value of rapid reviews in the context of emergent decision-making needs outweighs the disadvantages or risk of bias and potentially ‘imperfect' evidence. | 0.466 |
| 13 | Rapid reviews need to be tailored to the specific needs of the knowledge user. | 0.339 |
| 24 | It is important to have minimum standards for the methodological conduct of rapid reviews. | 0.298 |
| 9 | Rapid reviews do not replace SRs or HTAs. | 0.297 |
| 31 | Rapid reviews that omit an assessment of the quality of included studies are useless to knowledge users. | 0.296 |
| 33 | Transparency of process is more important than the actual methods used to produce rapid reviews, as transparency allows the end user to make their own assessment on validity and appropriateness. | 0.269 |
| 49 | Producers are more concerned with the methodology and validity of rapid reviews than knowledge users. | 0.235 |
| 23 | My confidence in a rapid review is directly tied to results being presented and contextualized by the strength and applicability of the evidence. | 0.211 |
| 16 | There is so much overlap across the various evidence synthesis methods that I cannot generalize my opinion to favor one over the other without the context of the decision at hand. | 0.15 |
| 8 | The opportunity cost of a comprehensive SR or HTA is too high and it is more advantageous to conduct rapid reviews when timeliness is a factor. | 0.059 |
| 26 | Standardization of rapid review methods may conflict with the needs of knowledge users | 0.011 |
| 48 | ‘Rapid review' is too broad a phrase – doing a review in a more timely way can only be relative to how long it takes the same team to produce a full systematic review. | -0.118 |
| 30 | Reporting of the results of rapid reviews must be tailored to the knowledge user(s) who commissioned the review. | -0.148 |
| 50 | It is difficult to judge the validity of a rapid review as the reporting is often truncated and protocols are not published. | -0.237 |
| 3 | Deviating from accepted systematic review methods may introduce bias and impact the validity of the resulting rapid review, which may be an unacceptable risk for some for knowledge users. | -0.262 |
| 34 | It is appropriate to endeavor to define a single, unique methodology for rapid reviews. | -0.393 |
| 37 | I put more confidence in evidence produced in a systematic review than of a rapid review. | -0.462 |
| 46 | It is difficult to tell a rapid review from a systematic review unless very specific nomenclature is used in the title or description of methods. | -0.48 |
| 44 | A rapid review must be justified with a valid rationale for both speeding up the process and tailoring rigourous methods for evidence synthesis. | -0.505 |
| 17 | There is a risk involved in tailoring accepted SR methods to produce rapid reviews that we do not yet understand. | -0.514 |
| 2 | When time allows, a comprehensive systematic review of all available evidence should always be conducted. | -0.783 |
| 42 | Any review of evidence that takes longer than 3 months to produce is not a rapid review. | -0.856 |
| 4 | Further research comparing the methods and results of rapid reviews and systematic reviews is required before I decide how I feel about rapid reviews. | -0.861 |
| 11 | Rapid reviews are comparable to SRs except they are done in a more timely fashion. | -0.893 |
| 15 | There is a paucity of evidence on rapid reviews, so I cannot support or oppose their use in decision-making. | -0.952 |
| 5 | Rapid reviews are too focused in scope and/or context to be generalizable to a variety of knowledge users. | -0.989 |
| 19 | It is always appropriate to conduct a rapid review. | -1.023 |
| 43 | Any review of evidence that takes longer than 1 month to produce is not a rapid review. | -1.396 |
| 47 | A rapid review cannot be a systematic review. | -1.412 |
| 40 | Rapid reviews should only be conducted when the alternate option is the use of no evidence to inform a decision. | -1.53 |
| 12 | Rapid reviews are ‘quick and dirty' systematic reviews. | -1.943 |
| 7 | Rapid reviews should only precede a more comprehensive and rigorous systematic review. | -2.135 |
| 38 | The more time spent conducting the review of the evidence, the more valid the results of the review will be. | -2.149 |
| **Factor B** | | |
| **#** | **Statements** | **Z-Score** |
| 3 | Deviating from accepted systematic review methods may introduce bias and impact the validity of the resulting rapid review, which may be an unacceptable risk for some for knowledge users. | 2.463 |
| 47 | A rapid review cannot be a systematic review. | 1.842 |
| 4 | Further research comparing the methods and results of rapid reviews and systematic reviews is required before I decide how I feel about rapid reviews. | 1.664 |
| 10 | All evidence synthesis products, including rapid reviews, SRs, or HTAs, can be conducted very well or very poorly. | 1.442 |
| 9 | Rapid reviews do not replace SRs or HTAs. | 1.242 |
| 13 | Rapid reviews need to be tailored to the specific needs of the knowledge user. | 1.021 |
| 36 | The results from a systematic review may not differ from those of a rapid review, but more research is needed to support this theory and quantify why results may be the same or different. | 0.843 |
| 6 | Rapid reviews mean different things to different people. | 0.843 |
| 2 | When time allows, a comprehensive systematic review of all available evidence should always be conducted. | 0.843 |
| 45 | A good quality review of evidence is determined by the methods used, not by the speed at which it is completed. | 0.821 |
| 21 | Appropriateness of a rapid review varies with the type of decision being made, and any financial, legal or other important contextual facets tied to the decision. | 0.621 |
| 11 | Rapid reviews are comparable to SRs except they are done in a more timely fashion. | 0.621 |
| 1 | The evidence from rapid reviews is good enough to inform low-risk, emergent policy or decision-making needs when the alternative is the use of no evidence. | 0.621 |
| 40 | Rapid reviews should only be conducted when the alternate option is the use of no evidence to inform a decision. | 0.421 |
| 37 | I put more confidence in evidence produced in a systematic review than of a rapid review. | 0.421 |
| 32 | Rapid reviews can be timely and valid, even when methodological concessions are made. | 0.421 |
| 24 | It is important to have minimum standards for the methodological conduct of rapid reviews. | 0.421 |
| 12 | Rapid reviews are ‘quick and dirty' systematic reviews. | 0.421 |
| 30 | Reporting of the results of rapid reviews must be tailored to the knowledge user(s) who commissioned the review. | 0.4 |
| 50 | It is difficult to judge the validity of a rapid review as the reporting is often truncated and protocols are not published. | 0.2 |
| 25 | It is important to have minimum standards for the reporting of rapid reviews (e.g., a PRISMA-RR). | 0.2 |
| 23 | My confidence in a rapid review is directly tied to results being presented and contextualized by the strength and applicability of the evidence. | 0.2 |
| 22 | My confidence in a rapid review is impacted by which methods are tailored to speed up the review process. | 0.2 |
| 16 | There is so much overlap across the various evidence synthesis methods that I cannot generalize my opinion to favor one over the other without the context of the decision at hand. | 0.2 |
| 14 | Rapid reviews meet the needs of knowledge users. | 0.2 |
| 46 | It is difficult to tell a rapid review from a systematic review unless very specific nomenclature is used in the title or description of methods. | 0 |
| 29 | Knowledge users do not fully understand the implications of streamlining evidence synthesis methods to produce a more timely evidence product. | 0 |
| 18 | Using rapid reviews to inform decisions is better than using no evidence at all. | 0 |
| 17 | There is a risk involved in tailoring accepted SR methods to produce rapid reviews that we do not yet understand. | 0 |
| 15 | There is a paucity of evidence on rapid reviews, so I cannot support or oppose their use in decision-making. | 0 |
| 49 | Producers are more concerned with the methodology and validity of rapid reviews than knowledge users. | -0.2 |
| 44 | A rapid review must be justified with a valid rationale for both speeding up the process and tailoring rigourous methods for evidence synthesis. | -0.2 |
| 41 | A well-conducted rapid review may produce better evidence than a poorly conducted systematic review. | -0.222 |
| 38 | The more time spent conducting the review of the evidence, the more valid the results of the review will be. | -0.222 |
| 20 | Rapid reviews and all other evidence synthesis products hold the same value as long as they retain the core value of being transparent in conduct, include the highest quality evidence available and present results with a qualification on the strength of evidence. | -0.222 |
| 48 | ‘Rapid review' is too broad a phrase – doing a review in a more timely way can only be relative to how long it takes the same team to produce a full systematic review. | -0.421 |
| 31 | Rapid reviews that omit an assessment of the quality of included studies are useless to knowledge users. | -0.421 |
| 39 | Achieving a precise estimate of effect (from a SR) may not inform the decision-at-hand any better than a general estimate of effect (produced by a rapid review). | -0.621 |
| 35 | Rapid reviews are not a unique methodology, they are simply a variation of a systematic review that can fall anywhere on the continuum of evidence synthesis methods. | -0.621 |
| 34 | It is appropriate to endeavor to define a single, unique methodology for rapid reviews. | -0.621 |
| 43 | Any review of evidence that takes longer than 1 month to produce is not a rapid review. | -0.821 |
| 27 | The value of rapid reviews in the context of emergent decision-making needs outweighs the disadvantages or risk of bias and potentially ‘imperfect' evidence. | -0.821 |
| 33 | Transparency of process is more important than the actual methods used to produce rapid reviews, as transparency allows the end user to make their own assessment on validity and appropriateness. | -0.843 |
| 26 | Standardization of rapid review methods may conflict with the needs of knowledge users | -1.242 |
| 5 | Rapid reviews are too focused in scope and/or context to be generalizable to a variety of knowledge users. | -1.242 |
| 42 | Any review of evidence that takes longer than 3 months to produce is not a rapid review. | -1.642 |
| 8 | The opportunity cost of a comprehensive SR or HTA is too high and it is more advantageous to conduct rapid reviews when timeliness is a factor. | -1.642 |
| 28 | Knowledge users don't always need all of the evidence, they just need the best evidence to support their decision, and what is ‘best evidence' is specific to the knowledge user. | -1.842 |
| 7 | Rapid reviews should only precede a more comprehensive and rigorous systematic review. | -2.263 |
| 19 | It is always appropriate to conduct a rapid review. | -2.463 |
| **Factor C** | | |
| **#** | **Statements** | **Z-Score** |
| 28 | Knowledge users don't always need all of the evidence, they just need the best evidence to support their decision, and what is ‘best evidence' is specific to the knowledge user. | 2.059 |
| 13 | Rapid reviews need to be tailored to the specific needs of the knowledge user. | 2.059 |
| 41 | A well-conducted rapid review may produce better evidence than a poorly conducted systematic review. | 1.373 |
| 33 | Transparency of process is more important than the actual methods used to produce rapid reviews, as transparency allows the end user to make their own assessment on validity and appropriateness. | 1.373 |
| 27 | The value of rapid reviews in the context of emergent decision-making needs outweighs the disadvantages or risk of bias and potentially ‘imperfect' evidence. | 1.373 |
| 24 | It is important to have minimum standards for the methodological conduct of rapid reviews. | 1.373 |
| 17 | There is a risk involved in tailoring accepted SR methods to produce rapid reviews that we do not yet understand. | 1.373 |
| 14 | Rapid reviews meet the needs of knowledge users. | 1.373 |
| 49 | Producers are more concerned with the methodology and validity of rapid reviews than knowledge users. | 0.686 |
| 46 | It is difficult to tell a rapid review from a systematic review unless very specific nomenclature is used in the title or description of methods. | 0.686 |
| 39 | Achieving a precise estimate of effect (from a SR) may not inform the decision-at-hand any better than a general estimate of effect (produced by a rapid review). | 0.686 |
| 32 | Rapid reviews can be timely and valid, even when methodological concessions are made. | 0.686 |
| 26 | Standardization of rapid review methods may conflict with the needs of knowledge users | 0.686 |
| 21 | Appropriateness of a rapid review varies with the type of decision being made, and any financial, legal or other important contextual facets tied to the decision. | 0.686 |
| 11 | Rapid reviews are comparable to SRs except they are done in a more timely fashion. | 0.686 |
| 8 | The opportunity cost of a comprehensive SR or HTA is too high and it is more advantageous to conduct rapid reviews when timeliness is a factor. | 0.686 |
| 6 | Rapid reviews mean different things to different people. | 0.686 |
| 3 | Deviating from accepted systematic review methods may introduce bias and impact the validity of the resulting rapid review, which may be an unacceptable risk for some for knowledge users. | 0.686 |
| 45 | A good quality review of evidence is determined by the methods used, not by the speed at which it is completed. | 0 |
| 44 | A rapid review must be justified with a valid rationale for both speeding up the process and tailoring rigourous methods for evidence synthesis. | 0 |
| 36 | The results from a systematic review may not differ from those of a rapid review, but more research is needed to support this theory and quantify why results may be the same or different. | 0 |
| 35 | Rapid reviews are not a unique methodology, they are simply a variation of a systematic review that can fall anywhere on the continuum of evidence synthesis methods. | 0 |
| 30 | Reporting of the results of rapid reviews must be tailored to the knowledge user(s) who commissioned the review. | 0 |
| 29 | Knowledge users do not fully understand the implications of streamlining evidence synthesis methods to produce a more timely evidence product. | 0 |
| 25 | It is important to have minimum standards for the reporting of rapid reviews (e.g., a PRISMA-RR). | 0 |
| 22 | My confidence in a rapid review is impacted by which methods are tailored to speed up the review process. | 0 |
| 20 | Rapid reviews and all other evidence synthesis products hold the same value as long as they retain the core value of being transparent in conduct, include the highest quality evidence available and present results with a qualification on the strength of evidence. | 0 |
| 18 | Using rapid reviews to inform decisions is better than using no evidence at all. | 0 |
| 10 | All evidence synthesis products, including rapid reviews, SRs, or HTAs, can be conducted very well or very poorly. | 0 |
| 9 | Rapid reviews do not replace SRs or HTAs. | 0 |
| 4 | Further research comparing the methods and results of rapid reviews and systematic reviews is required before I decide how I feel about rapid reviews. | 0 |
| 1 | The evidence from rapid reviews is good enough to inform low-risk, emergent policy or decision-making needs when the alternative is the use of no evidence. | 0 |
| 48 | ‘Rapid review' is too broad a phrase – doing a review in a more timely way can only be relative to how long it takes the same team to produce a full systematic review. | -0.686 |
| 42 | Any review of evidence that takes longer than 3 months to produce is not a rapid review. | -0.686 |
| 40 | Rapid reviews should only be conducted when the alternate option is the use of no evidence to inform a decision. | -0.686 |
| 37 | I put more confidence in evidence produced in a systematic review than of a rapid review. | -0.686 |
| 31 | Rapid reviews that omit an assessment of the quality of included studies are useless to knowledge users. | -0.686 |
| 23 | My confidence in a rapid review is directly tied to results being presented and contextualized by the strength and applicability of the evidence. | -0.686 |
| 19 | It is always appropriate to conduct a rapid review. | -0.686 |
| 16 | There is so much overlap across the various evidence synthesis methods that I cannot generalize my opinion to favor one over the other without the context of the decision at hand. | -0.686 |
| 15 | There is a paucity of evidence on rapid reviews, so I cannot support or oppose their use in decision-making. | -0.686 |
| 5 | Rapid reviews are too focused in scope and/or context to be generalizable to a variety of knowledge users. | -0.686 |
| 50 | It is difficult to judge the validity of a rapid review as the reporting is often truncated and protocols are not published. | -1.373 |
| 47 | A rapid review cannot be a systematic review. | -1.373 |
| 43 | Any review of evidence that takes longer than 1 month to produce is not a rapid review. | -1.373 |
| 38 | The more time spent conducting the review of the evidence, the more valid the results of the review will be. | -1.373 |
| 34 | It is appropriate to endeavor to define a single, unique methodology for rapid reviews. | -1.373 |
| 12 | Rapid reviews are ‘quick and dirty' systematic reviews. | -1.373 |
| 7 | Rapid reviews should only precede a more comprehensive and rigorous systematic review. | -2.059 |
| 2 | When time allows, a comprehensive systematic review of all available evidence should always be conducted. | -2.059 |

**Supplement 7‑17. Descending Array of Factor Differences**

**Factors A,B**

| **#** | **A** | **B** | **Delta** |
| --- | --- | --- | --- |
| 28 | 0.535 | -1.842 | 2.377 |
| 41 | 1.837 | -0.222 | 2.059 |
| 39 | 1.173 | -0.621 | 1.794 |
| 20 | 1.55 | -0.222 | 1.772 |
| 8 | 0.059 | -1.642 | 1.701 |
| 19 | -1.023 | -2.463 | 1.44 |
| 27 | 0.466 | -0.821 | 1.287 |
| 26 | 0.011 | -1.242 | 1.253 |
| 35 | 0.6 | -0.621 | 1.221 |
| 33 | 0.269 | -0.843 | 1.112 |
| 25 | 1.246 | 0.2 | 1.046 |
| 18 | 1.026 | 0 | 1.026 |
| 45 | 1.826 | 0.821 | 1.005 |
| 42 | -0.856 | -1.642 | 0.786 |
| 31 | 0.296 | -0.421 | 0.717 |
| 21 | 1.295 | 0.621 | 0.674 |
| 29 | 0.484 | 0 | 0.484 |
| 22 | 0.684 | 0.2 | 0.484 |
| 14 | 0.674 | 0.2 | 0.474 |
| 32 | 0.866 | 0.421 | 0.445 |
| 49 | 0.235 | -0.2 | 0.435 |
| 10 | 1.869 | 1.442 | 0.427 |
| 48 | -0.118 | -0.421 | 0.303 |
| 5 | -0.989 | -1.242 | 0.253 |
| 34 | -0.393 | -0.621 | 0.228 |
| 7 | -2.135 | -2.263 | 0.128 |
| 23 | 0.211 | 0.2 | 0.011 |
| 16 | 0.15 | 0.2 | -0.05 |
| 36 | 0.738 | 0.843 | -0.105 |
| 24 | 0.298 | 0.421 | -0.123 |
| 1 | 0.486 | 0.621 | -0.135 |
| 44 | -0.505 | -0.2 | -0.305 |
| 6 | 0.523 | 0.843 | -0.32 |
| 50 | -0.237 | 0.2 | -0.437 |
| 46 | -0.48 | 0 | -0.48 |
| 17 | -0.514 | 0 | -0.514 |
| 30 | -0.148 | 0.4 | -0.548 |
| 43 | -1.396 | -0.821 | -0.575 |
| 13 | 0.339 | 1.021 | -0.682 |
| 37 | -0.462 | 0.421 | -0.883 |
| 9 | 0.297 | 1.242 | -0.945 |
| 15 | -0.952 | 0 | -0.952 |
| 11 | -0.893 | 0.621 | -1.514 |
| 2 | -0.783 | 0.843 | -1.626 |
| 38 | -2.149 | -0.222 | -1.927 |
| 40 | -1.53 | 0.421 | -1.951 |
| 12 | -1.943 | 0.421 | -2.364 |
| 4 | -0.861 | 1.664 | -2.525 |
| 3 | -0.262 | 2.463 | -2.725 |
| 47 | -1.412 | 1.842 | -3.254 |
| **Factors A,C** | | | |
| **#** | **A** | **C** | **Delta** |
| 10 | 1.869 | 0 | 1.869 |
| 45 | 1.826 | 0 | 1.826 |
| 20 | 1.55 | 0 | 1.55 |
| 2 | -0.783 | -2.059 | 1.276 |
| 25 | 1.246 | 0 | 1.246 |
| 50 | -0.237 | -1.373 | 1.136 |
| 18 | 1.026 | 0 | 1.026 |
| 31 | 0.296 | -0.686 | 0.982 |
| 34 | -0.393 | -1.373 | 0.98 |
| 23 | 0.211 | -0.686 | 0.897 |
| 16 | 0.15 | -0.686 | 0.836 |
| 36 | 0.738 | 0 | 0.738 |
| 22 | 0.684 | 0 | 0.684 |
| 21 | 1.295 | 0.686 | 0.609 |
| 35 | 0.6 | 0 | 0.6 |
| 48 | -0.118 | -0.686 | 0.568 |
| 39 | 1.173 | 0.686 | 0.487 |
| 1 | 0.486 | 0 | 0.486 |
| 29 | 0.484 | 0 | 0.484 |
| 41 | 1.837 | 1.373 | 0.464 |
| 9 | 0.297 | 0 | 0.297 |
| 37 | -0.462 | -0.686 | 0.224 |
| 32 | 0.866 | 0.686 | 0.18 |
| 43 | -1.396 | -1.373 | -0.023 |
| 47 | -1.412 | -1.373 | -0.039 |
| 7 | -2.135 | -2.059 | -0.076 |
| 30 | -0.148 | 0 | -0.148 |
| 6 | 0.523 | 0.686 | -0.163 |
| 42 | -0.856 | -0.686 | -0.17 |
| 15 | -0.952 | -0.686 | -0.266 |
| 5 | -0.989 | -0.686 | -0.303 |
| 19 | -1.023 | -0.686 | -0.337 |
| 49 | 0.235 | 0.686 | -0.451 |
| 44 | -0.505 | 0 | -0.505 |
| 12 | -1.943 | -1.373 | -0.57 |
| 8 | 0.059 | 0.686 | -0.627 |
| 26 | 0.011 | 0.686 | -0.675 |
| 14 | 0.674 | 1.373 | -0.699 |
| 38 | -2.149 | -1.373 | -0.776 |
| 40 | -1.53 | -0.686 | -0.844 |
| 4 | -0.861 | 0 | -0.861 |
| 27 | 0.466 | 1.373 | -0.907 |
| 3 | -0.262 | 0.686 | -0.948 |
| 24 | 0.298 | 1.373 | -1.075 |
| 33 | 0.269 | 1.373 | -1.104 |
| 46 | -0.48 | 0.686 | -1.166 |
| 28 | 0.535 | 2.059 | -1.524 |
| 11 | -0.893 | 0.686 | -1.579 |
| 13 | 0.339 | 2.059 | -1.72 |
| 17 | -0.514 | 1.373 | -1.887 |
| **Factors B,C** | | | |
| **#** | **B** | **C** | **Delta** |
| 47 | 1.842 | -1.373 | 3.215 |
| 2 | 0.843 | -2.059 | 2.902 |
| 12 | 0.421 | -1.373 | 1.794 |
| 3 | 2.463 | 0.686 | 1.777 |
| 4 | 1.664 | 0 | 1.664 |
| 50 | 0.2 | -1.373 | 1.573 |
| 10 | 1.442 | 0 | 1.442 |
| 9 | 1.242 | 0 | 1.242 |
| 38 | -0.222 | -1.373 | 1.151 |
| 37 | 0.421 | -0.686 | 1.107 |
| 40 | 0.421 | -0.686 | 1.107 |
| 16 | 0.2 | -0.686 | 0.886 |
| 23 | 0.2 | -0.686 | 0.886 |
| 36 | 0.843 | 0 | 0.843 |
| 45 | 0.821 | 0 | 0.821 |
| 34 | -0.621 | -1.373 | 0.752 |
| 15 | 0 | -0.686 | 0.686 |
| 1 | 0.621 | 0 | 0.621 |
| 43 | -0.821 | -1.373 | 0.552 |
| 30 | 0.4 | 0 | 0.4 |
| 31 | -0.421 | -0.686 | 0.265 |
| 48 | -0.421 | -0.686 | 0.265 |
| 22 | 0.2 | 0 | 0.2 |
| 25 | 0.2 | 0 | 0.2 |
| 6 | 0.843 | 0.686 | 0.157 |
| 29 | 0 | 0 | 0 |
| 18 | 0 | 0 | 0 |
| 11 | 0.621 | 0.686 | -0.065 |
| 21 | 0.621 | 0.686 | -0.065 |
| 44 | -0.2 | 0 | -0.2 |
| 7 | -2.263 | -2.059 | -0.204 |
| 20 | -0.222 | 0 | -0.222 |
| 32 | 0.421 | 0.686 | -0.265 |
| 5 | -1.242 | -0.686 | -0.556 |
| 35 | -0.621 | 0 | -0.621 |
| 46 | 0 | 0.686 | -0.686 |
| 49 | -0.2 | 0.686 | -0.886 |
| 24 | 0.421 | 1.373 | -0.952 |
| 42 | -1.642 | -0.686 | -0.956 |
| 13 | 1.021 | 2.059 | -1.038 |
| 14 | 0.2 | 1.373 | -1.173 |
| 39 | -0.621 | 0.686 | -1.307 |
| 17 | 0 | 1.373 | -1.373 |
| 41 | -0.222 | 1.373 | -1.595 |
| 19 | -2.463 | -0.686 | -1.777 |
| 26 | -1.242 | 0.686 | -1.928 |
| 27 | -0.821 | 1.373 | -2.194 |
| 33 | -0.843 | 1.373 | -2.216 |
| 8 | -1.642 | 0.686 | -2.328 |
| 28 | -1.842 | 2.059 | -3.901 |

1. Fuerntratt, E. Zur Bestimmung der Anzahl interpretierbarer gemeinsamer Faktoren in Faktorenanalysen psychologischer Daten (The determination of the number of interpretable common factors in factor analysis of psychological data). Diagnostica. 1969;15:62–75.
